# Sexual Communication between a Dog and a Hen

**Published:** 1897-08

**Authors:** 


SEXUAL COMMUNICATION BETWEEN A DOG AND A HEN.—Cadiot, in the *Recueil de méd Vét*, reports the case of a dog that was accustomed to play with chickens. He gradually became accustomed to seize one of the hens and to carry out the act of coition. At first the hen resisted, but soon tried to provoke the act of coition, squatting before the dog and announcing its desire by a peculiar cackle, until coition followed. The hen was killed. —*Thierärzt. Centralblatt*, November 15, 1896.

